# Problematic Media Use and Anxiety Symptoms in Adolescents: The Role of Age and Gender

**DOI:** 10.3390/healthcare13030281

**Published:** 2025-01-31

**Authors:** Inmaculada Concepción Rodríguez-Rojo, Raquel Luengo-González, Cecilia Peñacoba-Puente, Montserrat García-Sastre, Ernesto Espín-Lorite, Daniel Cuesta-Lozano, Ángel Asenjo-Esteve, Concepción Noriega-Matanza

**Affiliations:** 1 Community Care and Social Determinants of Health (CUYDET), Nursing and Physiotherapy Department, Universidad de Alcalá, 28804 Alcalá de Henares, Spain; inmaculada.rr@uah.es (I.C.R.-R.); raquel.luengo@uah.es (R.L.-G.); ernesto.espin@uah.es (E.E.-L.); daniel.cuesta@uah.es (D.C.-L.); angel.asenjo@uah.es (Á.A.-E.); concha.noriega@uah.es (C.N.-M.); 2 Center for Cognitive and Computational Neuroscience (C3N), Universidad Complutense, 28040 Madrid, Spain; 3 Group for Research in Nursing Care, Gregorio Marañón, Health Research Institute (IiSGM), 28009 Madrid, Spain; 4 Psychology Department, Universidad Rey Juan Carlos, 28933 Madrid, Spain

**Keywords:** adolescence, problematic media use, anxiety, age, gender, moderating effect

## Abstract

Background: Adolescence is a critical biopsychosocial adjustment period, with increased susceptibility to problematic media use (PMU) and associated risk-taking behaviors. The aim of the present study consisted of identifying the relationship between PMU (i.e., videogames, mobile phones, internet, and television) and anxiety in adolescents, considering the moderating effect of gender and age. Methods: A descriptive study using a national survey was conducted on a sample of 4034 participants. Results: Significant gender mean differences were found in PMU and anxiety, with large and moderate effect sizes for PMU (videogames) in males (*d* = 0.86) and anxiety in females (*d* = 0.67). Additionally, a direct positive relationship between age and anxiety was observed. Furthermore, PMU significantly predicted anxiety after controlling for age and gender in all cases. Gender had a significant and stronger moderating effect on PMU (television) and anxiety for the male subgroup. The moderating effect of age on PMU and anxiety was statistically significant and more pronounced at younger ages. Conclusions: This research demonstrates the association between PMU and anxiety in adolescents, highlighting the need to further explore other moderating factors influencing mental health symptoms beyond age and gender. It is important to emphasize that mental health is a shared responsibility and not solely the domain of mental health professionals. Therefore, initiatives should be promoted to engage educators, parents, and policymakers in addressing this collective challenge.

## 1. Introduction

Adolescence is defined as a complex transitional stage between the ages of 10 and 24 that constitutes an increased susceptibility to brain circuitry maturation that could affect higher-order cognition and sociocultural development [1,2]. Of particular interest is the role of executive functions since they are essential to explaining psychosocial performance, mental health, or risk behavior throughout this period of life [3,4,5].

To ensure appropriate human identity formation in the adolescent (i.e., self-unity and self-esteem), it is crucial to be immersed in an environment rich in high-quality social interactions with both peers and family [6]. It should be noted that the spatial/physical distancing and staying at home measures to contain the spread and impact of COVID-19 dramatically limited adolescents’ opportunities for face-to-face contact [7]. Because of fear and uncertainty, the pandemic situation also precipitated worldwide mental health problems such as anxiety, depression, or stress [8,9], especially within the adolescent population [7,10].

Under this scenario, the advance and use of technology (i.e., internet, mobile devices, or applications) greatly facilitated accessibility to and the speed of sharing information, as well as maintaining remote social contact in different areas of everyday life. It has also been proven that young people reinforced their social network through online communication, which were particularly helpful in those cases where the adolescent manifests feelings of loneliness, depressive symptoms, or social anxiety [11,12]. Despite the current generation of adolescents growing up in a highly digitized society, the at-home confinement period prompted a breakdown in their daily routine and structure, which could even extend into the present day. To deal with this reality, recent research has reported the use of technology such as watching television (i.e., TV), playing videogames, surfing the internet, etc., as a potential coping or self-regulation mechanism that, in excess, could become problematic [13,14].

In this sense, problematic media use (PMU) refers to excessive engagement with screen-based media devices (such as computers, videogames, smartphones, tablets and television) or platforms (e.g., social media or online browsing), often involving disproportionate time spent on these activities [15]. This behavior is characterized by neglecting or replacing other essential tasks, such as completing assignments, engaging in face-to-face interactions, or participating in offline leisure and cultural activities. PMU could be also associated with “addiction-like behaviors” that disrupt children’s or adolescents’ personal, familial, academic, or social functioning, leading to potential negative consequences in their daily lives [16,17,18,19].

As already mentioned, adolescents are in a critical phase of their biopsychosocial development. Given this, it may be hypothesized that PMU patterns could be more frequently observed among young people with poorer mental health status, specifically manifesting symptoms related to anxiety and depression [7,18,20,21]. To understand this association, different studies have focused on the role of age and gender as mediating/moderating factors. For example, it has been established that younger adolescents (i.e., 14–15.9 years old), with heavier social networking site usages, are more likely to present internalizing behavior problems and lower academic competencies than older adolescents (i.e., 16–17.9 years old). The authors explained this result by arguing that the former have diminished social skills compared to the latter and are therefore more expected to suffer anger, depression, or a variety of behavioral/social alterations [22]. Regarding gender, Dong et al. [23] showed that females’ PMU (i.e., internet) was lower than that reported in males. A possible reason could be that compared to boys, girls mature earlier (both physically and psychologically) and are better in emotional regulation against stressful life events [24]. However, other studies have found opposite results, suggesting positive associations between age (i.e., junior vs. high school students), anxiety sensitivity, and smartphone PMU severity and between females and higher levels of depression [20] and females and smartphone PMU severity [25].

On the other hand, recent research carried out by Shannon and co-workers [18] found that when assessing the impact of age and gender on the relationship between PMU (specifically in relation to problematic social media use) and mental health outcomes (i.e., increased rates of depression, anxiety, and stress), neither of them had a moderating effect. Interestingly, a meta-review that analyzed the relationship between computer-mediated communication, social media, and mental health observed a small negative association between social network site use and mental health. Specifically, the authors argued that despite the literature supporting the contention that effects are influenced by mediators and moderators, meta-analyses provide limited evidence for these moderating effects regarding age and gender [26]. These inconsistencies could vary for reasons ranging from participants’ age range to the imbalance between female and male subgroups, the type of PMU, or even the conceptualization approach under consideration.

Bearing this in mind, the aim of the present study was to deepen our understanding of how different PMUs (i.e., videogames, mobile phones, internet, and TV) could impact adolescents’ mental health (i.e., anxiety), considering the moderating effect of age and gender. Potential hypotheses include the following:

**H1.**  *PMU is associated with higher levels of anxiety in adolescents, with the type of technology used influencing the strength of this relationship. It could be expected that platforms that promote social interactions (such as social media or online gaming) have a stronger impact on anxiety levels compared to those centered on passive content consumption (e.g., TV).*

**H2.**  *The relationship between PMU and anxiety levels in adolescents varies by age and gender. Younger adolescents are expected to show a stronger relationship between excessive technology use and anxiety compared to older adolescents due to their less-developed ability to cope with emotional impacts of digital interactions. Additionally, female adolescents (compared to male adolescents) are anticipated to experience higher levels of anxiety related to PMU due to differences in interaction styles and social expectations.*

## 2. Materials and Methods

A survey was designed and conducted throughout 2021. The research received approval from the Ethics Committee for Research in Health of the Universidad de Alcalá (Reg. CEIP/HU/2021/2/004). Adolescent assent and parents’ consent was obtained prior to participation in the study. The assent/consent process ensured that participants were fully aware of the study’s objectives, procedures, and their right to withdraw at any time without consequences. In line with ethical research standards, privacy and confidentiality were strictly maintained.

### 2.1. Participants

A total of 4034 participants filled the survey and, after removing those cases in which the data were incomplete, 4025 adolescents (52% females) from 12 to 18 years old (mean age 14.41 ± 1.74) were considered under study. They all were recruited from public and private secondary schools in Spain (see Table 1 for further information about the characteristics of the sample). Participants were asked to complete an online survey during the first six months of 2021 (14 March–21 June 2021). The survey included different questions regarding sociodemographic data, PMU, and the presence of anxiety symptoms (see the Instruments section for a more detailed description of these evaluation tools).

### 2.2. Instruments

An ad hoc online questionnaire was developed to collect sociodemographic variables such as age, sex, type of school, and city of origin. The following scales/questionnaires were also included.

#### 2.2.1. Generalized Anxiety Disorder Scale (GAD-7)

The GAD-7 questionnaire is a one-dimensional self-administered scale created to assess the presence of generalized anxiety disorder symptoms in adults [27], but it has also been validated in adolescents [28,29,30]. In this study, the Spanish version of García-Campayo et al. [31] was the one applied to the participants. The type of response consisted of a 4-point Likert-type scale from 0 to 3, where 0 meant not at all and 3 meant nearly every day. Thus, the GAD-7 scale scores ranged from 0 to 21, with 21 being the highest level of anxiety. The Cronbach’s alpha obtained for this variable in the present study was 0.88.

#### 2.2.2. Problematic Use of New Technologies Questionnaire

This questionnaire was created and validated as a tool for assessing PMU in children and adolescents (specifically, internet, videogames, mobiles, and TV). It comprises 41 items. The first two are related to the frequency of use and potential problems when using different new technologies. Additionally, it incorporates 10 other items concerning the use of the internet, videogames, and mobile phones, and 9 extra items associated with TV use [32]. All questions are closed-ended with a range of possible answers. Furthermore, the questionnaire includes items on frequency of use, existing problems, and characteristics of use such as location and hours of use. In this case, the Cronbach’s alphas for the sample under study were 0.78 for videogame use, 0.67 for internet use, 0.79 for mobile phone use, and 0.76 for TV use.

### 2.3. Statistical Analysis

In the first place, for the variables of interest, descriptive statistics and internal consistency (measured by Cronbach’s alpha) were calculated. A Student’s *t*-test was performed to determine the mean differences between the variables (PMU and anxiety) for male and female genders. In addition, Pearson correlations were conducted among all the variables. Second, eight multivariate regressions were carried out using the PROCESS macro (model 1) [33], with four regressions for gender as a moderator and four for age as a moderator. Each regression included a combination of the independent variable (PMU), the moderators (age and gender), and their interaction to predict the outcome (anxiety). Post hoc analyses were conducted when a significant moderation was found to examine the effects of the independent variables on the outcome at different levels of the moderator. Finally, an alpha level of 0.05 was set for all the analyses conducted using SPSS version 22.

## 3. Results

### 3.1. Differences in Problematic Media Use (PMU) and Anxiety According to Gender

Table 2 represents significant gender mean differences for PMU variables and anxiety (all *p* < 0.001, except for PMU (TV), *p* = 0.008). More specifically, women had higher scores in PMU (mobile phone), PMU (internet), and (anxiety), while men showed higher scores in PMU (videogames). However, and despite obtaining highly significant *p*-values, when considering Cohen’s *d*, only PMU (videogames) and anxiety had, respectively, large (*d* = 0.86) and medium (*d* = 0.67) effect sizes.

### 3.2. Descriptive Data and Correlations Among Variables

As can be seen in Table 3, significant positive correlations were observed between PMU and anxiety (all *p* < 0.001 except for PMU (videogames); r = 0.612). In relation to age, significant but weak correlations were observed between age and PMU (mobile phone) (r = 0.05; *p* < 0.001) and PMU (internet) (r = 0.08; *p* < 0.001), and between age and PMU (videogames) (r = −0.11; *p* < 0.001) and PMU (TV) (r = −0.05; *p* < 0.001). Finally, a significant positive correlation was observed between anxiety and age (r = 0.12; *p* < 0.001).

### 3.3. Multivariate Associations and Moderation Analyses

#### 3.3.1. Gender as Moderator

After controlling for age, the results from the multivariate hierarchical regression analyses that predicted anxiety from PMU, gender, and their interaction are displayed in Table 4. The results indicate a positive main effect of PMU on all models, with the greatest impact being from PMU (internet). In all the models, gender had a significant direct negative effect on anxiety, meaning that women had higher scores than men.

One moderation effect of gender was found in the relationship between PMU (TV) and gender on anxiety (β = 0.225; t = 1.92; *p* = 0.049; 95% CI = −0.005, 0.456).

As noted in Table 5, in men (regarding women), the presence of anxiety symptoms will further depend on PMU (TV).

#### 3.3.2. Age as Moderator

After controlling for gender, the results of the multivariate hierarchical regression analyses used to forecast anxiety from PMU, age, and their interaction are shown in Table 6. All models revealed a positive main effect of PMU, with the most significant impact being for PMU (internet). In all of the models, age had a significant positive direct effect on anxiety.

Indeed, three moderation effects of age were found, as follows: (a) regarding the relationship between PMU (mobile phone) and age on anxiety (β = −0.030; t = −2.34; *p* = 0.019; 95% CI = −0.055, −0.005); (b) in the relationship between PMU (videogames) and age on anxiety (β = −0.046; t = −2.01; *p* = 0.044; 95% CI = −0.09, −0.001); and (c) in the association between PMU (internet) and age on anxiety (β = −0.079; t = −3.17; *p* = 0.001; 95% CI = −0.127, −0.030). The highest effect size observed was in the relationship between PMU (internet) and age of anxiety.

As evidenced in Table 7, in all cases, the younger the age, the greater the relationship between PMU (i.e., mobile phone, videogames, and internet) and anxiety.

## 4. Discussion

The aim of the present study consisted of identifying the relationship between PMU (i.e., videogames, mobile phone, internet, and TV) and the presence of anxious symptomatology in a sample of adolescents between 12–18 years old. Moreover, the moderating effects of gender and age were also considered to better elucidate this type of association.

As already mentioned, adolescence is a crucial period of life where cognitive and socioaffective development takes place [1,2]. These processes are accompanied by the maturation of brain regions that are important for executive functioning (i.e., planning, decision making, behavioral control, or social understanding and communication), like, for example, the prefrontal cortex [34,35]. Because of this biopsychosocial period of adjustment, adolescents are considered, amongst other things, to be more susceptible to impulsivity or risk-taking behaviors, which, in turn, could lead to greater “addictive practices”, such as a PMU [3,36]. If we then consider that this population is also immersed in a media-saturated world where social interactions mainly occur throughout the use of technologies, it is necessary to determine which adolescents could be more/less vulnerable to beneficial or undesirable media influences. In this regard, Achterberg and co-workers [37] followed longitudinal associations between structural brain development, social media use, and mental health. Their results demonstrated different brain trajectories linked to media use and mental health, setting a foundation to examine which adolescents may benefit from social media/technologies use, and who might be negatively affected.

On the other hand, it is well known that the COVID-19 pandemic introduced changes in adolescents’ routines and that they also experienced moderate-to-high levels of anxiety and other physical and psychological disorders [7,10,38,39]. Additionally, and compared to pre-pandemic and post-pandemic rates, children and adolescents have manifested increased exposure time to electronic screen devices (i.e., TV, digital media, videogames, and e-learning), which, ultimately, has been strongly associated with behavioral problems, depression, anxiety, distress, and low well-being [22,40,41,42,43].

Since the outcomes could vary depending on age and gender, there is a growing interest in contemplating the effect of these two variables. For instance, one pre-pandemic study observed a significant gender-related association between time of exposure to videogames and anxiety, with this relation being positive for adolescent females but negative for males [44]. Others have found positive associations between age, anxiety sensitivity, and smartphone PMU severity [20], or differences between initial levels of problematic internet use related to gender, which, in turn, could be modulated by perceived loneliness [45]. However, there are very few studies that have explored the relationship between PMU and anxiety when controlled for gender and age. In this sense, our results went one step further and evidenced that after adjusting for both variables, PMU contributed to higher levels of anxiety, and this effect was particularly significant for PMU (internet). This specific outcome could be due to the popularity and easy accessibility to this technology. Nevertheless, despite its benefits (i.e., exchange and availability of information at any time and any place), it has been proven that frequent/excessive visits to websites, chats, social media platforms, etc., could negatively impact on adolescents’ mental health [46,47].

Another interesting result from our investigation was that related to the direct effect of gender on anxiety, even when PMU was controlled. This effect was observed for every technology under study (i.e., videogames, mobile phone, internet, and TV), and, in all cases, females presented more anxiety symptoms than males. This is consistent with our second hypothesis and other studies that examined differences in anxiety at two time points: before the COVID-19 pandemic (T1) and two months after government restrictions and online learning were introduced (T2). The authors observed that compared to males, females not only evidenced a greater level of anxiety at T1 but also a significant increase in anxious symptomatology from T1 to T2 [48]. Similarly, a cross-national investigation of 566,829 adolescents across 73 countries confirmed that, overall, females have greater levels of mental health disorders than males [49]. While the exact cause could oscillate from biological to environmental factors, some research suggests that puberty is a time in females’ life marked by hormonal fluctuations and a predisposition to psychosocial stress, which make them more likely to experience anxiety and mood disorders than their male counterparts [50].

Alternatively, when comparing PMU scores by gender, our results (accounting for the effect size and the large sample size of our study) revealed that males exhibited increased scores associated with PMU (videogames). This might be explained by the fact that males’ preferences are more directly linked to gaming and electronic devices in general, while females spend more time on smartphones, social media (e.g., for communicating with friends), general computer use, and online [47,51,52]. Similarly, German research on problematic gaming (i.e., computer games) in youth (mean age = 14.16 years) and its association with different dimensions of quality of life, exhibited that male sex, younger age, reduced physical activity, and poorer school performance were linked to greater gaming severity [53]. As a possible explanation of this result, it could be hypothesized that within the videogame environment, male adolescents have the power to easily control and establish interpersonal contacts that could be more complex for them in the context of face-to-face interactions [54].

Looking at the moderating effect of gender between PMU and anxiety, and contrary to what we have proposed in our first hypothesis (i.e., that adolescents should show lower levels of anxiety when using devices centered on more passive content consumption, such as TV), our results demonstrated a significant effect of gender in its interaction with PMU (TV) in the explanation of anxiety. The post hoc analysis indicated that the effect of PMU (TV) on anxiety was greater for men. It is noteworthy that time spent watching TV during childhood and adolescence has been associated with an increased risk of being diagnosed with an anxiety disorder during early and middle-life adulthood [55]. What is not completely clear is whether the use of technology, that is, watching TV by adolescent males, is employed as a coping/self-regulation mechanism rather than the cause of anxiety by itself. Given that, research carried out by Boursier et al. [56] observed that for the general population, the increase in watching TV series during the COVID-19 lockdown could probably serve as a recovery strategy in facing such a stressful situation. Regarding females, it has been evidenced that they might be more prone to relying on their social networks or social support when dealing with life stressors [57,58,59].

As well as gender, our findings indicated the influence of age on the variables under study (PMU and anxiety). Specifically, and considering the effect sizes, significant correlations of interest were observed between PMU (videogames) and age, indicating that the older the age, the lower the PMU (videogames). It is probable that videogames (and TV content) might be more familiar, accessible, appealing, or adapted to younger adolescents’ needs [60] than the telephone or the internet. Moreover, as occurred with gender, our results showed a direct effect of age on anxiety levels, even when controlling for PMU. In the same vein, this outcome was observed for the PMU of the four technologies considered in the study. In all cases, there was a positive association between age and anxiety. This result was supported by other investigations where adolescents had higher anxiety rates compared to pre-adolescents [61]. It has been stated that adolescence is a period of strong sensitivity to peer influence. Following brain maturation process and executive function development during this stage of life, it could be said that older adolescents may become more self-conscious of their social responsibilities. This awareness can lead to anxiety-related symptoms or “addiction-like behaviors” (i.e., PMU) if they struggle to manage personal, academic, or social roles appropriately. In this sense, Somerville and co-workers [62] examined the association between the developmental modulation of socioaffective brain regions (i.e., medial prefrontal and striatum-medial prefrontal cortex connections) and adolescents’ preoccupations concerning how they are perceived by others. They found that adolescents’ self-conscious emotion was higher than that of children and adults (i.e., the age of peak embarrassment rating was 17.2 years). In addition, acute self-consciousness has been linked to anxiety disorders among adolescents [63,64].

With respect to the moderating effect of age in the effect of PMU on anxiety, three statistically significant effects were observed (i.e., PMU (mobile phone), PMU (videogames), and PMU (internet)), with the PMU–anxiety association being more pronounced at younger ages, as we had speculated in our second hypothesis. This could lead us to assume that at older ages, anxiety would depend on other factors such as the PMU. The maturation process of the adolescent’s brain, to which we previously referred, is also associated with the awareness of other concerns that can cause anxiety, such as those indicated by Magson and collaborators (i.e., their inability to see their friends, friends or family members becoming seriously ill or dying from COVID-19, or the impossibility to participate in social life or activities) [48]. Whether these situations could directly affect young or older adolescents is something that should be assessed in the near future. Hence, it is essential to do an in-depth study of the risk and protective factors that could prevent mental health and PMU, especially at the earliest stages of adolescence.

### Limitations and Future Directions

The present research is limited by some potential weaknesses. For example, it employed a cross-sectional design and a correlational approach, so we cannot draw firm conclusions about the causal pathways involved. Future studies should consider the implementation of longitudinal studies to investigate all these aspects and their potential interactions, particularly in contexts different from those experienced after the COVID-19 pandemic. Similarly, future research should explore the bidirectional relationship between PMU and anxiety, investigating how both factors influence each other over time, while also considering the moderating effects of variables beyond age and gender (i.e., personality, family dynamics, economic status, social support, stressful situations, media exposure and/or social connection, the neural mechanisms that may be implicated, etc.).

Furthermore, it should be taken into account that in questionnaire-based studies, there might be a gender gap per se; this a product of reporting bias, as males are often less willing to report their negative mental health status than females. Another aspect related to questionnaires has to do with the fact that despite considering a continuous approach when measuring PMU, introducing cut-off points or creating categories of PMU could have served as other viable approximations, particularly for identifying clinical or risk thresholds. Future investigations should explore and validate these methods to better distinguish between typical and problematic usage patterns.

On the other hand, while the large sample size in this study is a significant strength, ensuring the representativeness and generalizability of our findings, it may also introduce a methodological bias. Specifically, large samples can yield statistically significant results for effect sizes that are small and may have limited clinical significance. Future research should aim to balance sample size considerations with efforts to assess the practical implications of the findings in an everyday context.

In addition to all the aforementioned methodological discrepancies, it is also essential to recognize the broader conceptual diversity in the field. Many studies on the relationship between technology use and mental health rely on varying definitions and indicators, such as “screen time” or mental health markers like self-esteem, loneliness, or depression. As demonstrated by recent meta-reviews [26], this inconsistency not only arises from differences in study populations but also from the conceptualization and operationalization of the constructs themselves. This highlights the importance of establishing more solid theoretical and methodological frameworks for a more accurate and comparative assessment of effects in different populations and contexts, moving beyond technology-centered approaches (e.g., time spent, frequency) to the features of communication channels, types of interactions/dynamics, or message content.

Finally, considering that mental health impacts all domains and is not confined solely to the field of mental health professionals, in what follows, we list several possible recommendations for educators, parents, and policymakers to make our findings more actionable.

In the case of educators, it is essential to promote digital literacy in the classroom by designing activities focused on the responsible use of technologies. These activities should consider the associated potential risks when carrying out excessive use, especially in the early stages of adolescence. Furthermore, fostering emotional well-being in school is imperative. Designing programs that explain what mental health is and the importance of mental health self-care, particularly for female adolescents, can be highly beneficial.

For parents, it could be interesting to establish screen time limits, especially for younger adolescents. This can be achieved by supervising the adolescents’ use of technology, setting clear schedules, and encouraging the development of alternative activities (i.e., sports, family time). Additionally, it is crucial to promote effective communication between parents and their children. This includes discussing how they feel when using devices, the potential risks, establishing adequate parenting styles, and providing emotional support to help them when dealing with anxiety or other mental health issues. This support is especially important for adolescent females as they present a greater vulnerability.

Regarding policymakers, it should be necessary to implement strategies combining educational programs and regulations to promote responsible media use in schools. This can contribute to increasing competence-based pedagogical approaches, bridging the digital divide, and ensuring equitable access to technology, while training educators to guide safe and effective use. Moreover, family and community programs that support balanced media use and mental health resources should be developed, providing training for parents and educators on privacy protection, internet safety, and digital citizenship, while also offering tools to manage device use and set clear limits at home.

## 5. Conclusions

Given the intrinsic characteristics of adolescence and the occurrence of the COVID-19 pandemic, it is essential to highlight the importance of exploring its psychological effects on the emotional well-being of adolescents. As a result, this study contributes to the current body of research by emphasizing the relationship between PMU and adolescents’ mental health.

It is important to consider that the variation in the relationship between PMU and anxiety across age groups, as well as its moderation by gender, holds significant educational and clinical implications. Recognizing these differences is essential for designing targeted interventions and guiding professionals in mental health, educators, parents, and policymakers in developing strategies with which to identify vulnerable groups, mitigate negative effects of PMU, and ensure appropriate media use and emotional well-being.

## Figures and Tables

**Table 1:** Characteristics of the sample (n = 4025).

Age Frequencies (Number of Participants and Percentage)						
12 years	13 years	14 years	15 years	16 years	17 years	18 years
586; 14.6%	891; 22.1%	773; 19.2%	592; 14.7%	540; 13.4%	470; 11.7%	173; 4.2%
**Gender**						
Female				Male		
2092; 52%				1933; 48%		
**Type of school**						
Public				Private		
2751; 68.4%				1273; 31.6%		
**Region/Area**						
Rural				Urban		
529, 13.1%				3496; 86.9%		

**Table 2:** Gender mean differences in problematic media use (PMU) and anxiety.

	Female	Male	*p*	Cohen’s *d*
PMU mobile phone	10.4 ± 3.5	9.6 ± 3.2	<0.001	0.23
PMU videogames	4.5 ± 1.3	6.3 ± 2.3	<0.001	0.86
PMU TV	4.5 ± 1.1	4.6 ± 1.6	0.008	0.08
PMU internet	5.6 ± 1.8	5.4 ± 1.7	<0.001	0.15
Anxiety	9.0 ± 5.6	5.4 ± 4.5	<0.001	0.67

Values are presented as mean ± standard deviation. PMU: problematic media use; TV: television.

**Table 3:** Means, standard deviations, and Pearson correlations among variables.

	Mean	SD	2	3	4	5
1. PMU mobile phone	9.99	3.36	0.26 **	0.39 **	0.65 **	0.36 **
2. PMU videogames	5.39	2.06		0.30 **	0.31 **	0.61
3. PMU TV	4.58	1.38			0.29 **	0.14 **
4. PMU internet	5.50	1.78				0.27 **
5. Anxiety	7.24	5.37				

SD: standard deviation; PMU: problematic media use; TV: television; ** *p* < 0.001.

**Table 4:** Prospective prediction of anxiety from problematic media use (PMU), gender, and their interaction.

	*R* ^2^	*F*	*p*	Beta	*t*	*p*	95% CI
DV = Anxiety	0.223	289.24	<0.001				
PMU mobile phone				0.509	22.72	<0.001	0.465, 0.553
Gender				−3.14	−20.91	<0.001	−3.44, −2.85
Interaction				−0.05	−1.12	0.263	−0.138, 0.037
Age				0.281	6.55	<0.001	0.197, 0.365
DV = Anxiety	0.154	183.49	<0.001				
PMU videogames				0.540	10.75	<0.001	0.441, 0.638
Gender				−4.48	−25.25	<0.001	−4.83, −4.13
Interaction				−0.070	−0.72	0.471	−0.262, 0.121
Age				0.384	8.55	<0.001	0.296, 0.472
DV= Anxiety	0.147	173.33	<0.001				
PMU TV				0.568	9.54	<0.001	0.451, 0.685
Gender				−3.60	−22.99	<0.001	−3.91, −3.29
Interaction				0.225	1.92	0.049	−0.005, 0.456
Age				0.344	7.66	<0.001	0.256, 0.432
DV = Anxiety	0.180	221.07	<0.001				
PMU internet				0.720	12.37	<0.001	0.606, 0.834
Gender				−3.46	−6.94	<0.001	−4.44, −2.48
Interaction				0.019	0.224	0.822	−0.15, 0.18
Age				0.263	5.97	<0.001	0.177, 0.350

CI: confidence interval; DV: dependent variable; PMU: problematic media use; TV: television.

**Table 5:** Conditional effects of problematic media use (PMU) (TV) on anxiety depending on gender.

Gender	Beta (PMU TV)	*t*	*p*	95% CI
Female	0.459	4.89	<0.001	0.275, 0.644
Male	0.685	9.70	<0.001	0.547, 0.824

CI: confidence interval; PMU: problematic media use; TV: television.

**Table 6:** Prospective prediction of anxiety from problematic media use (PMU), age, and their interaction.

	*R* ^2^	*F*	*p*	Beta	*t*	*p*	95% CI
DV = Anxiety	0.224	290.60	<0.001				
PMU mobile phone				0.945	5.06	<0.001	0.579, 1.31
Age				0.578	4.30	<0.001	0.315, 0.841
Interaction				−0.030	−2.34	0.019	−0.055, −0.005
Gender				−3.13	−20.84	<0.001	−3.43, −2.84
DV = Anxiety	0.155	184.53	<0.001				
PMU videogames				1.172	3.58	<0.001	0.530, 1.81
Age				0.624	4.88	<0.001	0.373, 0.875
Interaction				−0.046	−2.01	0.044	−0.09, −0.001
Gender				−4.45	−25.79	<0.001	−4.79, −4.11
DV = Anxiety	0.147	172.58	<0.001				
PMU TV				1.092	2.33	0.019	0.175, 2.01
Age				0.502	3.22	0.001	0.197, 0.808
Interaction				−0.034	−1.05	0.293	−0.098, 0.029
Gender				−3.61	−22.99	<0.001	−3.91, −3.29
DV = Anxiety	0.182	224.14	<0.001				
PMU internet				1.87	5.16	<0.001	1.16, 2.58
Age				0.701	4.85	<0.001	0.418, 0.984
Interaction				−0.079	−3.17	0.001	−0.127, −0.030
Gender				−3.35	−21.78	<0.001	−3.65, −3.05

CI: confidence interval; DV: dependent variable; PMU: problematic media use; TV: television.

**Table 7:** Conditional effects of problematic media use (PMU) with respect to mobile phones, videogames, and the internet on anxiety, organized by age.

Age	Beta (PMU mobile phone)	*t*	*p*	95% CI
13	0.554	19.15	<0.001	0.497, 0.611
14	0.524	22.78	<0.001	0.479, 0.569
16	0.464	15.45	<0.001	0.405, 0.522
**Age**	**Beta (PMU videogames)**	***T***	***p***	**95% CI**
13	0.575	11.45	<0.001	0.477, 0.674
14	0.529	12.52	<0.001	0.446, 0.612
16	0.438	7.46	<0.001	0.323, 0.553
**Age**	**Beta (PMU internet)**	**t**	***p***	**95% CI**
13	0.845	14.94	<0.001	0.734, 0.956
14	0.766	17.15	<0.001	0.678, 0.853
16	0.608	10.58	<0.001	0.495, 0.720

CI: confidence interval; PMU: problematic media use.

## Data Availability

The data have not been uploaded to a public repository. The data will be made available upon request to the corresponding authors.
